# Coexistence of congenital diaphragmatic hernia and abdominal wall closure defect with chromosomal abnormality: two case reports

**DOI:** 10.1186/s13256-016-0805-y

**Published:** 2016-01-22

**Authors:** Seiichiro Inoue, Akio Odaka, Yuki Muta, Yoshifumi Beck, Hisanori Sobajima, Masanori Tamura

**Affiliations:** 1 Department of Hepato-Biliary-Pancreatic Surgery and Pediatric Surgery, Saitama Medical Center, Saitama Medical University, Kamoda 1981, Kawagoe, Saitama 3508550 Japan; 2 Department of Neonatology, Center for Maternal, Fetal and Neonatal Medicine, Saitama Medical Center, Saitama Medical University, Kamoda 1981, Kawagoe, Saitama 3508550 Japan

**Keywords:** Abdominal wall closure defect, Chromosomal abnormality, Congenital diaphragmatic hernia

## Abstract

**Background:**

We reported two rare cases of congenital diaphragmatic hernia with abdominal wall closure defect, which were not associated with septum transversum diaphragmatic defects or Fryns syndrome.

**Case presentation:**

Case 1: a Japanese baby boy was delivered at 37 weeks’ gestation by urgent cesarean section because of the diagnosis of severe fetal distress. Congenital diaphragmatic hernia with omphalocele was prenatally diagnosed with fetal ultrasound. A ruptured omphalocele was confirmed at delivery. A silo was established on the day of his birth; direct closure of his diaphragmatic defect and abdominal wall closure was performed on the fifth day after his birth. Trisomy 13 was confirmed by genetic examination. His postoperative course was uneventful and he was discharged 5 months postnatally with home oxygen therapy. He was readmitted because of heart failure and died at 6 months.

Case 2: a Japanese baby boy, who was prenatally diagnosed with gastroschisis, was delivered at 35 weeks’ gestation by urgent cesarean section because of the diagnosis of fetal distress. Silo construction using a wound retractor was performed on the day of his birth and direct abdominal closure was performed on the tenth day after his birth. Trisomy 21 was confirmed by genetic examination. Treatment for his respiratory distress was continued after surgery. A retrosternal hernia was revealed at 6 months and direct closure of retrosternal diaphragm with the resection of hernia sac was performed. His postoperative course was uneventful and he was discharged with home oxygen therapy.

**Conclusions:**

Attention should be paid to chromosomal abnormality in cases in which the coexistence of congenital diaphragmatic hernia and abdominal wall closure defect are observed.

## Background

Malformations associated with congenital diaphragmatic hernia (CDH) can influence the morbidity and prognosis of the affected neonate [1]. The combination of CDH and an abdominal wall defect (omphalocele or gastroschisis) is rare; some babies with this combination have been reported to have a poor prognosis [2, 3]. This combination can be associated with syndromes [4, 5], but cases in which the etiology is unclear have also been reported [2, 3].

We encountered two cases of CDH combined with an abdominal wall defect. This report describes their clinical features, which are unlike those reported previously, and our surgical management. Our two patients did not fit the syndromes previously reported, but both had chromosomal anomalies.

## Case presentation

### Case 1

**Fig. 1:**
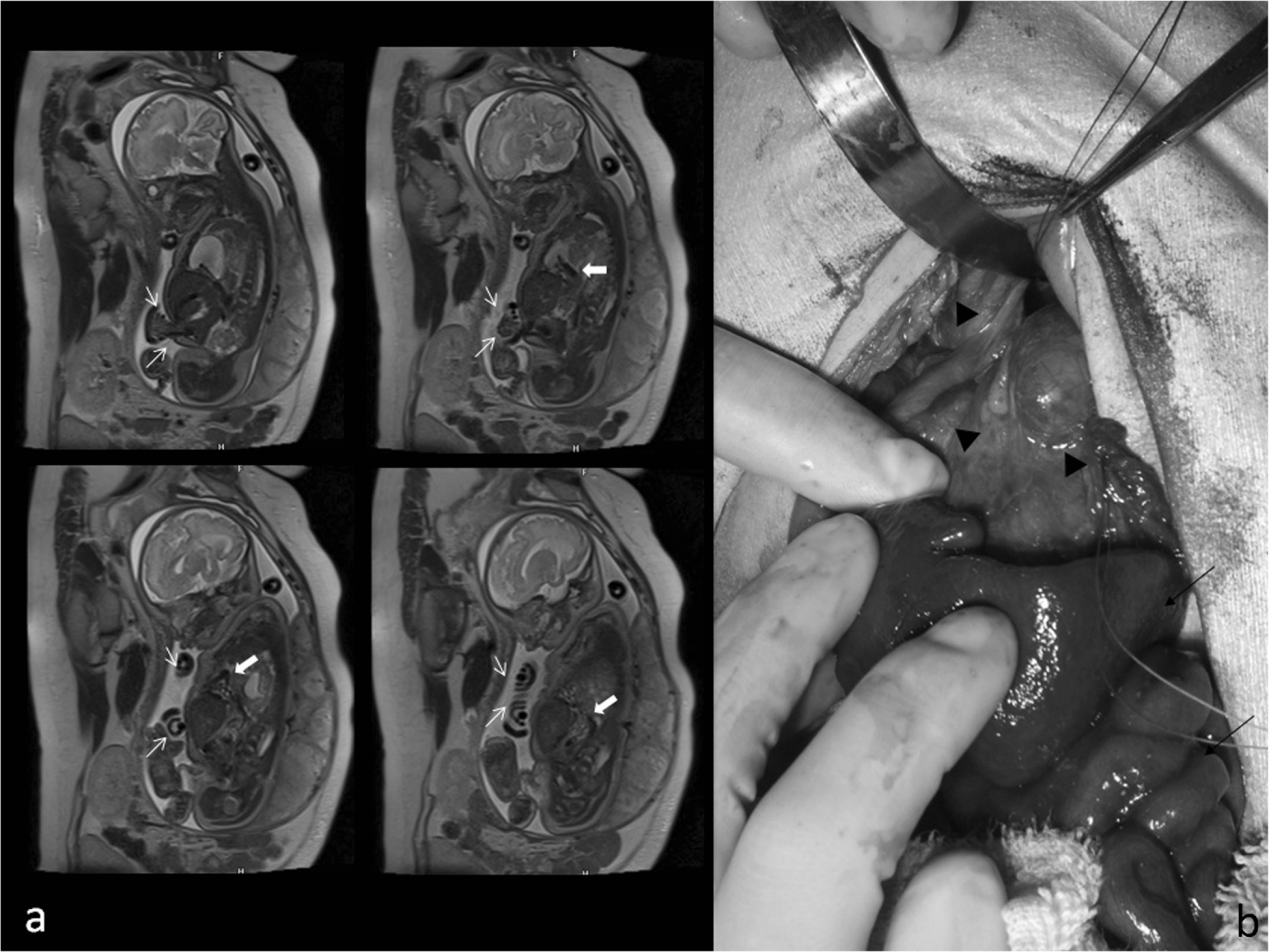
Fetal magnetic resonance imaging (**a**) and photograph at surgery (**b**) of Case 1. **a** Herniation of abdominal organs into the left pleural cavity (➩) and intestine into the umbilical cord (↓) were detected by magnetic resonance imaging at 34 weeks of gestation. **b** The spleen and small intestine were removed from the left pleural cavity and reduced into the abdomen (↓). Anterior, posterior, and left lateral posterior sides of diaphragm were formed but mediastinal side, especially around the esophageal hiatus of diaphragm, was defective (▼)

A Japanese baby boy was delivered at 37 weeks’ gestation by emergent cesarean section because of the diagnosis of severe fetal distress. At 33 to 34 weeks’ gestation, fetal CDH and omphalocele had been detected by fetal ultrasonography and magnetic resonance imaging (MRI; Fig. 1a). Immediately after delivery, he was intubated and mechanical ventilation was started. His birth body weight was approximately 2700 g. His Apgar score was four at 1 minute and eight at 5 minutes. Left CDH was confirmed by radiography. Multiple associated malformations (coarctation of aorta, left ventricular hypoplasia, left multicystic kidney, micropenis, right anophthalmia and left microphthalmia) were observed. Trisomy 13 was suspected and the diagnosis was confirmed by genetic testing.

Construction of a silo using a wound retractor [6] was urgently performed on the first day of his life because his hernial sac ruptured at delivery. The defect size of his abdominal wall was approximately 3×4 cm diameter. His entire small intestine and two thirds of his colon were outside his abdominal cavity. After his condition was stabilized, repair of the diaphragmatic hernia and closure of his abdominal wall were successfully performed on day 5. A hernial sac containing his spleen, two thirds of his stomach, and his polycystic left kidney had entered his chest cavity through a 2×2 cm defect in his diaphragm. The posterior, anterior, and lateral borders of the defect consisted of diaphragmatic muscle, but the medial border was formed by the esophageal hiatus (Fig. 1b). Direct closure of the defect was performed after resection of the hernial sac and then his abdominal wall was also closed primarily.

A postoperative surgical complication, such as wound infection or recurrence of CDH, was not observed. Although his respiratory and circulative conditions were unstable, his parents did not allow surgical treatment for coarctation of aorta and left ventricular hypoplasia. Conservative intensive treatments were performed by a neonatologist. Mechanical ventilation with intratracheal intubation was continued until day 22 after birth; then, nasal continuous positive airway pressure (CPAP) support was performed because hypoxia, which might have been due to congenital cardiovascular disease and apnea due to trisomy 13, was observed. He was discharged 5 months postnatally with home oxygen therapy. However, he was readmitted because of heart failure and died at 6 months.

### Case 2

**Fig. 2:**
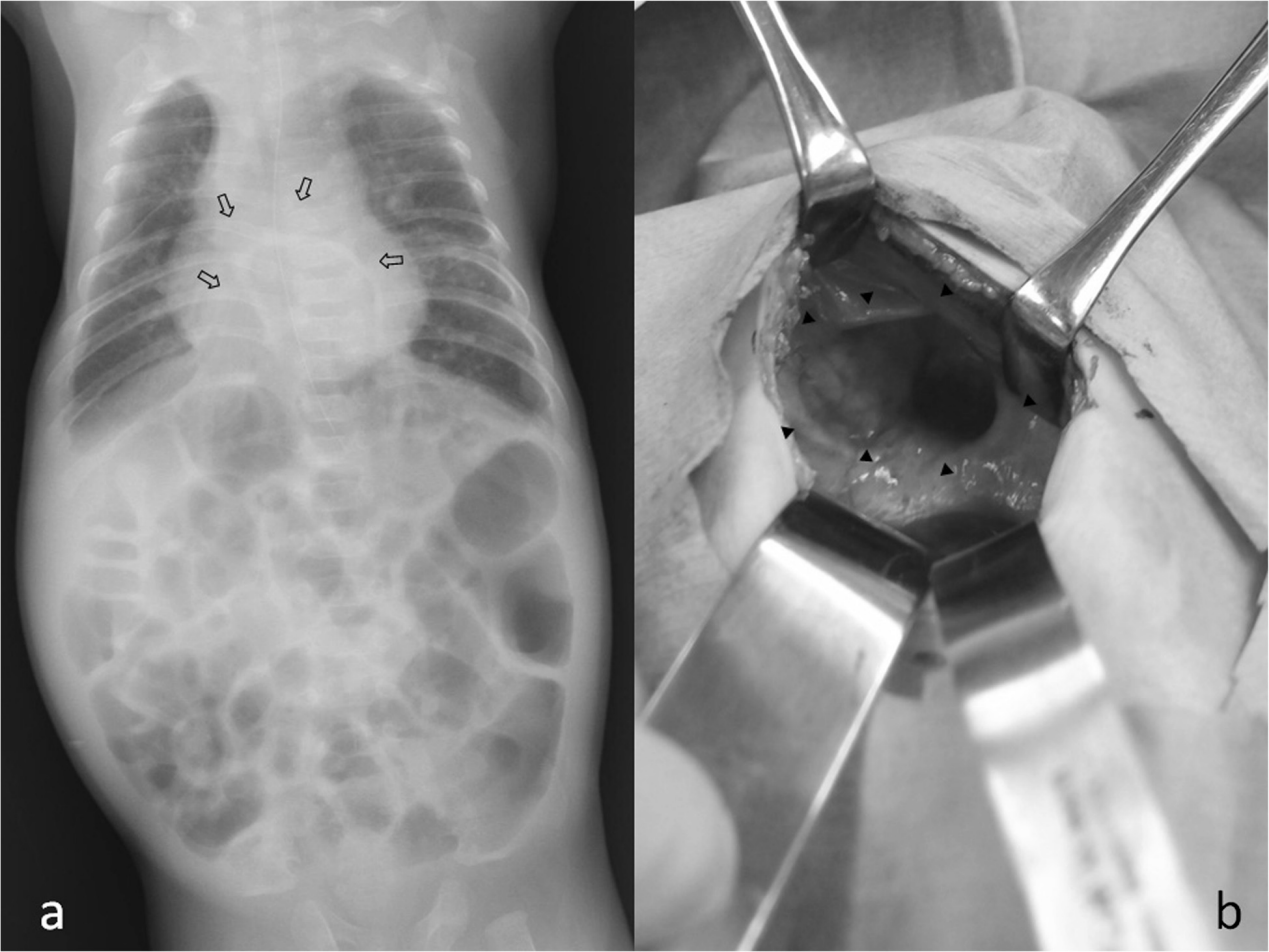
Radiograph on day 161 of life (**a**) and photograph during direct closure of the diaphragm (**b**) in Case 2. **a** On day 161, intestinal gas was detected in the mediastinum (➩). **b** A retrosternal hernial orifice (▼) was observed at operation and the defect was closed directly

A Japanese baby boy was delivered at 35 weeks’ gestation by emergent cesarean section because of the diagnosis of fetal distress. At 21 weeks’ gestation, fetal gastroschisis had been detected by fetal ultrasonography. Five minutes after delivery, he was intubated and mechanical ventilation was started because of hypoxia. His birth weight was 1892 g and his Apgar score was seven at 1 minute and eight at 5 minutes. His entire small intestine, part of his colon, and the greater curvature of his stomach were outside his abdomen, so a silo was constructed using a wound retractor [6]. He had severe generalized edema and hypovolemia, requiring intensive management. On day 10 of his life, direct closure of his abdominal defect was performed. After silo establishment, severe systemic edema and intravenous hypovolemia were observed and intensive neonatal care was performed by a neonatologist. Although he recovered from abdominal distention soon after abdominal closure, his respiratory condition was unstable and intensive care was continued. Trisomy 21 was confirmed by genetic examination. Mechanical ventilation with intratracheal intubation was continued until day 16 after birth, and then nasal CPAP respiratory support was performed because of his prolonged hypercapnea which might have been due to trisomy 21. Chest X-ray examinations were performed periodically. On day 161 after his birth, intestinal gas in his mediastinum was detected radiographically (Fig. 2a), although his respiratory condition was unchanged. Direct closure of a retrosternal defect in his diaphragm measuring 2.5×3.5 cm (Fig. 2b) was performed along with resection of his hernial sac. His postoperative course was uneventful and he was discharged.

## Discussion

Coexistence of CDH and an abdominal wall defect is considered to be rare and our case 2 is the first report of gastroschisis associated with a retrosternal hernia. Borys and Taxy performed a 10-year review of autopsy records and reported that the incidence of CDH associated with omphalocele was 0.077 % [7], while others have reported that the incidence of CDH combined with an abdominal wall defect is 1.16 % [1], 1.87 % [8], and 3.1 % [9]. In 2006, Harmath *et al*. reported a 9 to 11 % incidence of CDH associated with an abdominal wall defect (gastroschisis in two out of 71 cases and omphalocele in four cases) that was higher than the frequencies given in the reports just cited [10]. Coexistence of CDH with abdominal defects has previously been reported in neonates with a septum transversum diaphragmatic defect, which leads to midline anomalies such as the pentalogy of Cantrell or CDH plus a large epigastric omphalocele without sterna or cardiac anomalies, or neonates who have omphalocele associated with a congenital intrapericardial diaphragmatic hernia [4, 11, 12]. CDH is also associated with abdominal wall defects in Fryns syndrome: an autosomal recessive syndrome that features multiple congenital anomalies including distal digital hypoplasia, coarse facies, abnormalities of the ears, and CDH [5, 13]. Coexistence of CDH with abdominal wall defects has also been reported in non-syndromic patients. In these patients, the diaphragmatic defect was not a typical left posterolateral Bochdalek defect, but was found on the right side [14] or in the left anterolateral region [2], or else the intestine was seen in the right thorax with bilateral hypoplastic lungs on fetal magnetic resonance imaging [3]. Our two cases were considered to be non-syndromal because both did not have a typical Bochdalek defect in the diaphragm (the medial border of the defect was formed by the esophageal hiatus in case 1 and case 2 had a retrosternal defect). However, both of our patients had chromosomal abnormalities (trisomy 13 in case 1 and trisomy 21 in case 2). Harmath *et al*. reported the association of CDH and omphalocele in a patient with trisomy 18 [10], so an accumulation of more cases is needed for further analysis of the coexistence of CDH and abdominal wall defects.

Other anomalies associated with CDH may have an important influence on survival and it was recently reported that pulmonary hypoplasia is critical [2, 3]. Accurate diagnosis of both CDH and the abdominal wall defect is required because the timing of surgical treatment for CDH is important. In patients with both anomalies, underestimation of CDH or overestimation of the abdominal cavity volume can occur. There have been reports on diagnosis of CDH after abdominal wall closure or of CDH causing symptoms such as severe respiratory distress or circulatory instability after the abdominal wall defect is treated [11, 13, 14]. In our case 1, accurate prenatal diagnosis allowed successful treatment by establishing a silo at delivery, followed by surgical closure of the defects in the diaphragm and abdominal wall after stabilization of the patient’s general condition. In case 2, CDH was asymptomatic at delivery and emerged after approximately 6 months. Thus, the position of the defect in the diaphragm also affects the clinical course.

## Conclusions

We treated two cases of CDH associated with an abdominal wall defect. Coexistence of these anomalies is rare and may be associated with septum transversum diaphragmatic defects or Fryns syndrome. Our cases did not feature such anomalies, but both babies had chromosomal abnormalities. In neonates with abdominal wall defects and chromosomal abnormalities, we need to pay attention to the possible coexistence of CDH (which may be concealed). Accumulation of more cases is required to determine whether this rare combination is due to multiple malformation syndromes and defects of midline development or occurs by chance.

## Consent

Written informed consent was obtained from the parents of the patients for publication of this case report and any accompanying images. A copy of the written consents is available for review by the Editor-in Chief of this journal.

## Abbreviations

CDH: congenital diaphragmatic hernia
CPAP: continuous positive airway pressure
